# Studies of the Tarragon Essential Oil Effects on the Characteristics of Doped Hydroxyapatite/Chitosan Biocomposites

**DOI:** 10.3390/polym15081908

**Published:** 2023-04-16

**Authors:** Daniela Predoi, Simona Liliana Iconaru, Carmen Steluta Ciobanu, Mariana Stefania Raita, Liliana Ghegoiu, Roxana Trusca, Monica Luminita Badea, Carmen Cimpeanu

**Affiliations:** 1 National Institute of Materials Physics, Atomistilor Street, No. 405A, 077125 Magurele, Romania; 2 Faculty of Veterinary Medicine, University of Agronomic Sciences and Veterinary Medicine of Bucharest, 105 Splaiul Independentei, District 5, 050097 Bucharest, Romania; 3 Science and Engineering of Oxide Materials and Nanomaterials, Faculty of Applied Chemistry and Materials Science, University POLITEHNICA of Bucharest, Gh. Polizu 1-7, 011061 Bucharest, Romania; 4 National Center for Micro and Nanomaterials, University POLITEHNICA of Bucharest, Splaiul Independentei 313, 060042 Bucharest, Romania; 5 Faculty of Horticulture, University of Agronomic Sciences and Veterinary Medicine, 59 Marasti Blvd., 011464 Bucharest, Romania; 6 Faculty of Land Reclamation and Environmental Engineering, University of Agronomic Sciences and Veterinary Medicine of Bucharest, 59 Marasti Blvd, 011464 Bucharest, Romania

**Keywords:** zinc, hydroxyapatite, tarragon essential oil, chitosan, morphology, biocompatibility, antimicrobial activity

## Abstract

Due to the emergence of antibiotic-resistant pathogens, the need to find new, efficient antimicrobial agents is rapidly increasing. Therefore, in this study, we report the development of new biocomposites based on zinc-doped hydroxyapatite/chitosan enriched with essential oil of *Artemisia dracunculus* L. with good antimicrobial activity. Techniques such as scanning electron microscopy (SEM), X-ray diffraction (XRD), energy dispersive X-ray spectroscopy (EDX) and Fourier transform infrared spectroscopy (FTIR) were used in order to evaluate their physico-chemical properties. Our studies revealed that biocomposite materials with nanometric dimension and homogeneous composition could be obtained through an economic and cost-effective synthesis method. The biological assays demonstrated that ZnHA (zinc-doped hydroxyapatite), ZnHACh (zinc-doped hydroxyapatite/chitosan) and ZnHAChT (zinc-doped hydroxyapatite/chitosan enriched with essential oil of *Artemisia dracunculus* L.) did not exhibit a toxic effect on the cell viability and proliferation of the primary osteoblast culture (hFOB 1.19). Moreover, the cytotoxic assay also highlighted that the cell morphology of the hFOB 1.19 was not altered in the presence of ZnHA, ZnHACh or ZnHAChT. Furthermore, the in vitro antimicrobial studies emphasized that the samples exhibited strong antimicrobial properties against *Escherichia coli* ATCC 25922, *Staphylococcus aureus* ATCC 25923 and *Candida albicans* ATCC 10231 microbial strains. These results are encouraging for the following development of new composite materials with enhanced biological properties that could promote the osteogenic process of bone healing and also exhibit good antimicrobial properties.

## 1. Introduction

Lately, the unwanted effects of excessive and uncontrolled use of antibiotics in the curing of various infectious diseases have become more and more evident [1,2]. Thus, an exponential evolution of infectious diseases determined by multi-resistant pathogens was observed, these being difficult to treat, involving higher cost, and becoming an important public health problem [1]. Last but not least, they lead to the alteration of the patients’ quality of life. Recently, according to the results of the studies reported by Thompson T. [2], in 2019, over 1.2 million people died due to the diseases developed as a result of antimicrobial resistance. In this context, in order to ameliorate this public health problem, sustained efforts are being made to find new cheap and effective antimicrobial agents [3,4,5]. Hydroxyapatite (HA) is one of the commonly used bioceramics in bone tissue engineering, due to its outstanding biological features and similar composition with bone tissue (HA) [6,7]. However, a major disadvantage of hydroxyapatite is its weak antimicrobial activity. Therefore, in order to confer good antimicrobial properties, into the hydroxyapatite structure may be introduced metallic ions, such as zinc, cerium, silver, etc. [8,9,10,11,12]. Among these, zinc ions play an important role in the bone physiology [13] involved in processes such as bone development and DNA replication [7]. In the study made by P. Kazimierczak et al. [14], it was reported that zinc-doped hydroxyapatite biomaterials may support the bone regeneration process and even prevent the occurrence of post-surgery infections due to their outstanding biological properties [14]. Previously, it has been reported that both zinc (0.01 < x_Zn_ < 0.05) and chitosan (variation of chitosan concentration between 10 and 20 %wt) concentration play a key role in defining the antimicrobial properties of biomaterials [8,9,15]. In their study, A. Rasyida and collaborators [15] showed that the best antibacterial activity of Zn^2+^ doped HA in chitosan matrix against gram-negative and gram-positive bacterial strains was obtained for a concentration of 10% of chitosan [15]. Moreover, it was shown that the presence of copper and zinc ions in the chitosan/HA scaffolds provided them with good antibacterial activity (against *Escherichia coli* DH5α and *Staphylococcus aureus*) and also with improved osteoproliferative features [16]. Furthermore, Predoi D. et al. [17] highlighted that zinc and silver co-doped hydroxyapatite in chitosan matrix (coatings and suspension) do not alter the morphology or proliferation of the hFOB 1.19 cell line. Additionally, in our previous studies, it was reported that zinc and silver co-doped hydroxyapatite in chitosan matrix exhibited a good antimicrobial activity against the *Candida albicans* ATCC 90029, *Escherichia coli* ATCC 25922, and *Staphylococcus aureus* ATCC 25923 microbial strains [17].

Other methods, used for the obtaining of desired antimicrobial features, are represented by the incorporation of HA/doped HA in a biopolymeric matrix (such as chitosan, starch, and cellulose) [18] and/or adding natural compounds (e.g., essential oils (EO)) in the HA synthesis process [18]. Tarragon (*Artemisia dracunculus* L.) is a perennial subshrub member of the Asteraceae family [19]. Tarragon is generally used as a spice, but it also possesses antimicrobial, hepatoprotective and antioxidant properties [19,20,21]. In previous studies conducted by Fildan, A.P. and collaborators [21], it was underlined that the main components of tarragon essential oils are represented by estragole, limonene, methyleugenol, sabinene, β-ocimene, trans-anethole, α-ocimene, terpinolene, elemicin and terpinen-4-ol [21]. In the studies made by A. Farsanipour et al. [22], it was reported that by coating the *Scomberoides commersonnianus* muscle fillets with chitosan, whey protein isolated and tarragon essential oil were improved, and both the antibacterial activity and antioxidant features of the packaging material were improved. In another study, conducted by Zedan H. and coworkers [23], the authors reported that by adding a concentration of 6% chitosan (high molecular weight) and 20% of tarragon essential oil in yogurt, yogurts with higher shelf life and improved consistency could be obtained.

Taking into consideration all of these important features, the synthesis of a new biocomposite based on Zn^2+^ doped HA in chitosan matrix with essential oil of *Artemisia dracunculus* L. is of great interest to the bioengineering field. The proposed aim of this work was to obtain, through a cost-effective method (coprecipitation), new biocomposite materials based on zinc-doped hydroxyapatite/chitosan enriched with essential oil of *Artemisia dracunculus* L. Further, the physico-chemical features of the obtained biocomposite materials were analyzed by scanning electron microscopy (SEM), X-ray diffraction (XRD), energy dispersive X-ray spectroscopy (EDX) and Fourier transform infrared spectroscopy (FTIR). The in vitro biocompatibility of ZnHA (zinc-doped hydroxyapatite), ZnHACh (zinc-doped hydroxyapatite/chitosan), and ZnHAChT (zinc-doped hydroxyapatite/chitosan with essential oil of *Artemisia dracunculus* L.) was evaluated using a primary osteoblast culture (hFOB 1.19). Finally, the antimicrobial activity of ZnHA, ZnHACh and ZnHAChT against *Escherichia coli* ATCC 25922, *Staphylococcus aureus* ATCC 25923 and *Candida albicans* ATCC 10231 microbial was investigated.

## 2. Materials and Methods

### 2.1. Materials

The synthesis of zinc-doped hydroxyapatite (Ca_10−x_Zn_x_(PO_4_)_6_(OH)_2_ with x_Zn_ = 0.06) powder in chitosan matrix (ZnHApCh) enriched with essential oil of *Artemisia dracunculus* L. (ZnHAChT) was obtained through the coprecipitation method using calcium nitrate tetrahydrate, ammonium hydroxide, zinc nitrate hexahydrate of higher purity (≥99.0%), diammonium acid phosphate, chitosan (C_12_H_24_N_2_O_9_, low molecular weight: 50.000–190.000 Da; ≥75% (deacetylated)), and absolute ethanol procured from Sigma Aldrich, St. Louis, MO, USA. The essential oil of *Artemisia dracunculus* L. was purchased from Florame, Saint Rémy de Provence, France.

Zinc-doped hydroxyapatite suspensions in chitosan with tarragon essential oil were obtained by way of the coprecipitation technique [17,24]. The [Ca + Zn]/P ratio was 1.67. The solutions of Ca(NO_3_)_2_∙4H_2_O and Zn(NO_3_)_2_ 6H_2_O together with tarragon essential oil (3 mL in 100 mL H_2_O) were dropped into the solution of (NH_4_)_2_∙HPO_4_ and C_6_H_11_NO_4_. The pH was maintained constant at 10 throughout the drip. Then, the resulting suspension was stirred continuously for 6 h, after which it was centrifuged and redispersed. The washing procedure was performed 5 times. Subsequent to the last wash, the final precipitate was redispersed in a solution of 1% chitosan and tarragon essential oil (2 mL in 100 mL H_2_O) followed by continuous stirring for 12 h. The resulting final suspension (ZnHAChT) was evaluated both from a biological and physico-chemical point of view.

### 2.2. Characterization Methods

#### 2.2.1. Physico-Chemical Characterization

In order to measure the crystal size in the ZnHAp, ZnHACh and ZnHAChT samples, their X-ray diffraction patterns were measured. The patterns were achieved using a Rigaku SmartLab 3 kW (Rigaku, Tokyo, Japan) diffractometer with Kα Cu radiation (λ = 1.5418 Å) [24]. The incidence angle was 0.5° [24]. The patterns were recorded in the 2θ range 20–60°, with an angle variation of 0.02°, and a detector data acquisition total time of 8.5 s. The crystallite sizes of the samples were obtained by using Scherrer’s equation [25]:

L = Kλ/β·cosθ

where L is nano crystallite size, K is dimensionless shape factor, λ (nm) is X-ray wavelength from measuring full width at half maximum of peaks (β) in radian located at any 2θ in the pattern [26].

A scanning electron microscope (FEI Quanta Inspect F) equipped with an energy dispersive X-ray (EDX) device was used to analyze the morphology of samples. For the SEM studies, the powder was placed on a double-adhesive carbon tape without being subsequently covered with Au. The particle size distribution was also obtained from the SEM micrographs. Absorbance FTIR spectra of samples were collected on a SP 100 Perkin Elmer FTIR spectrometer (Waltham, MS, USA). The parameters used for the spectrum acquisition have been described elsewhere [24].

#### 2.2.2. In Vitro Antimicrobial Assay

The antimicrobial properties of the ZnHA, ZnHACh and ZnHAChT samples were assessed in vitro with the aid of reference *Staphylococcus aureus* ATCC 25923 (ATCC, Old Town Manassas, VA, USA), *Escherichia coli* ATCC 25922 (ATCC, Old Town Manassas, VA, USA) and *Candida albicans* ATCC 10231 (ATCC, Old Town Manassas, VA, USA) microbial strains. The in vitro experiments were performed in accordance with the method previously described in [27,28]. The assays were conducted using 0.5 McFarland standard microbial cultures. For this purpose, the biocomposites were inoculated with a volume of 1.5 mL microbial suspension having a cell density of 5 × 10^6^ CFU/mL (colony forming units/mL), prepared in phosphate-buffered saline (PBS), and afterwards incubated for 24, 48 and 72 h. A free microbial culture was also assessed for 24, 48 and 72 h and used as a positive control (C+). Suspensions of each culture medium were collected at different time intervals (24, 48 and 72 h) and incubated on a LB agar medium for 24 h at 37 °C. The number of CFU/mL was determined for each of the incubated samples with the microbial suspensions. The experiments were performed in triplicate and the data was presented as mean ± SD.

#### 2.2.3. Cytotoxicity Assay

The biocompatible properties of the ZnHA, ZnHACh and ZnHAChT were determined using a primary osteoblast culture (hFOB 1.19) prepared following the protocol devised by Gallagher et al. [29]. The in vitro cell viability experiments were conducted using the methodology previously described in [30]. For this purpose, for the growth and development of hFOB 1.19 cells, DMEM (Dulbecco’s Modified Eagle’s Medium) enriched with L-glutamine, sodium pyruvate, non-essential amino acids, sodium bicarbonate and fetal bovine serum was used. Afterwards, the cell cultures were incubated in a 5% CO_2_ atmosphere at a temperature of 37 °C. After reaching confluence, the culture was treated with trypsin, DMEM medium with 10% trypsin-inhibiting fetal serum was added to the cell suspension in trypsin and then the medium was centrifuged for 3 min at 1500 rpm. The cells were resuspended in a minimal volume of medium (500 µL), counted and distributed equally in 3–4 culture plates. The cells were then seeded in Petri plates and incubated with the samples at 37 °C in an atmosphere having 5% CO_2_. After 24 h, the cells were visualized under an inverted microscope using an Olympus IX71 microscope (Olympus, Tokyo, Japan). For the microscopic evaluation, the cultures were stained using phalloidin-FITC. For the cell viability assay, the cells were treated with MTT solution [3-(4,5dimethylthiazolyl)-2,5-diphenyltetrazolium bromide] and incubated in an atmosphere with 5% CO_2_ at 37 °C, and the optical density of the solubilized formazan at 595 nm was determined using a TECAN spectrophotometer.

#### 2.2.4. Statistical Analysis

The biological assays were performed in triplicate. For the statistical analysis, the t-test and analysis of variance (ANOVA) was used. The difference established between specimens was appreciated to be significant for a value of *p* < 0.05.

## 3. Results

**Figure 1:**
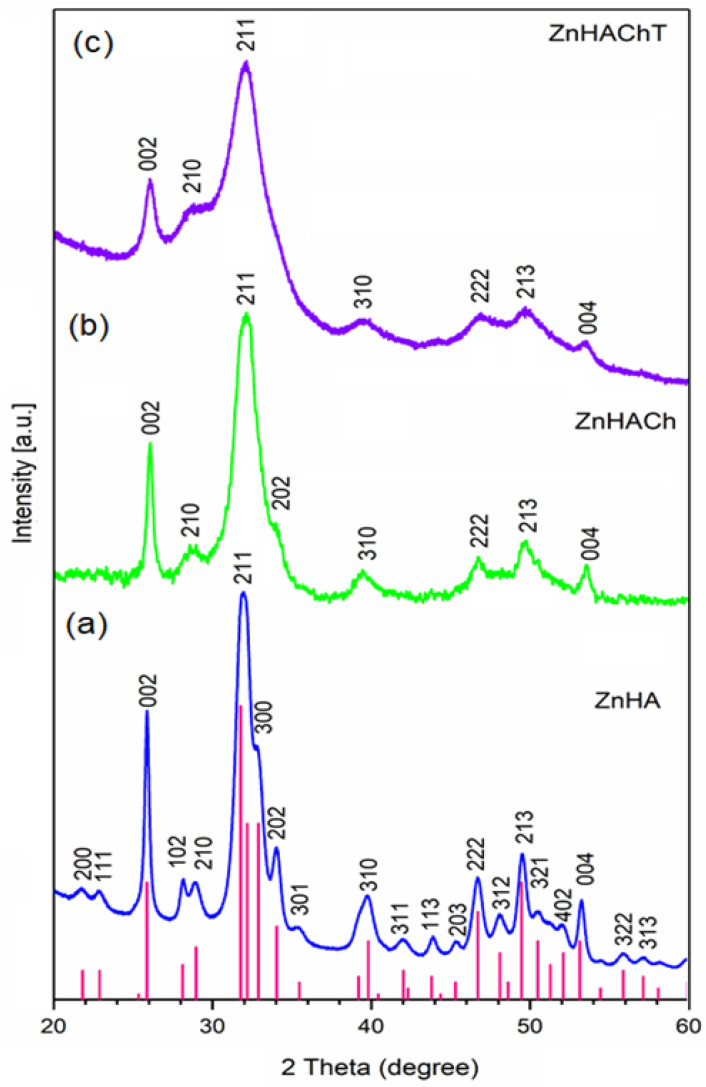
X-ray diffractograms of ZnHA (**a**), ZnHACh (**b**) and ZnHAChT (**c**) powders, and pattern for the reference hexagonal structure JCPDF 9-432 (red lines).

Figure 1 shows the XRD patterns for ZnHAp, ZnHACh and ZnHAChT. The diffraction patterns of ZnHAp and ZnHACh samples are similar to the pattern for pure hydroxyapatite with the reference hexagonal structure JCPDF 9-432 [24]. In agreement with previous studies [31], in the analyzed ZnHAp and ZnHApCh samples, a single phase of HA was identified. The presence of chitosan led to a slight shift of the peaks to larger angles. The crystallites of the ZnHACh sample are smaller than the crystallites of the ZnHA sample. This fact can also be observed from the width of the diffraction maxima. This behavior is in accord with previous studies [32]. The presence of the essential oil of *Artemisia dracunculus* led to a structural disturbance in the case of the ZnHAChT sample. The diffraction maxima of the ZnHAChT sample corresponds to the reference HA model but are much wider. In this case, the width of the diffraction maxima is due both to the crystallite size, which is smaller than in the case of the ZnHA and ZnHACh samples. The part of the material that becomes amorphous could lead to the formation of a calcium deficient HA.

The crystallite sizes of ZnHA, ZnHACh and ZnHAChT samples were 18 nm, 14 and 7 nm, respectively. It is noticed that the size of the particles decreased along with the modification of their surface.

The size of the crystallites was the smallest in the case of the ZnHAChT sample in which an amorphous phase is also present. Our results are in concordance with the studies on “Effect of dilute gelatine on the ultrasonic thermally assisted synthesis of nano-hydroxyapatite” previously reported by Brundavanam, R.K. et al. [33] when gelatine was used.

**Figure 2:**
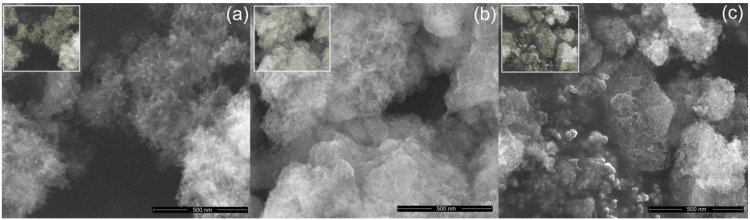
SEM images of the ZnHA (**a**), ZnHACh (**b**) and ZnHAChT (**c**) samples at 100,000× magnification and inset of measured particles used to determine particle size.

The SEM micrographs of the samples with ZnHA, ZnHACh and ZnHAChT are presented in Figure 2. The ZnHA sample has an acicular morphology (Figure 2a). In the case of samples synthesized in the presence of chitosan (ZnHACh), the morphology is slightly modified, tending towards the ellipsoidal shape (Figure 2b). The ZnHAChT (Figure 2c) sample in which, in addition to chitosan, the EO of *Artemisia dracunculus* L. is also present, has two types of morphologies. There are particles with an almost spherical morphology, but also particles that are not well-defined, forming different agglomerated formations that seem not to be well crystallized. This result supports the XRD studies which, in addition to the phase of HA, also highlighted an amorphous phase, in the case of the ZnHAChT sample. In addition to the change in morphology, a variation in the size of the particles was also observed. It is noticed that the particle size decreases from ZnHA to ZnHAChT. As can be observed for of all the studied samples, there is a tendency for the particles to agglomerate. In agreement with previous studies on the “synthesis of nano hydroxyapatite powder that simulate teeth particle morphology and composition” [34] and “biodegradation and bioresorption of calcium phosphate ceramics” [35] the particles tend to aggregate as a result of the small volume and due to the fact that the area/volume ratio is greater than in the case of particles on a submicron or micro scale.

**Figure 3:**
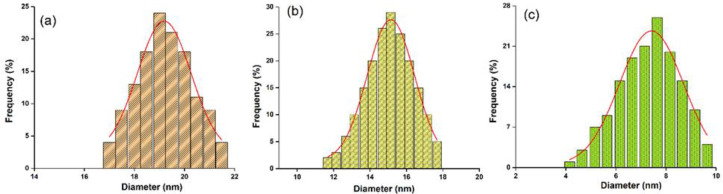
Size distributions of the ZnHA (**a**), ZnHACh (**b**) and ZnHAChT (**c**) samples. The red lines were obtained by fitting the experimental data with a Gaussian function.

The size distributions of the ZnHA, ZnHACh and ZnHAChT samples, which were obtained by counting a number of approximately 400 particles, are presented in Figure 3. It can be observed that the average size in the case of the ZnHA sample was 19 ± 1 nm, while for the ZnHACh sample, it was 15 ± 1 nm. The size distribution in the case of the ZnHAChT sample was obtained by counting the particles with almost spherical morphology, obtaining an average size of 7 ± 2 nm. Particles that are not well-defined and form agglomerated formations were not measured.

**Figure 4:**
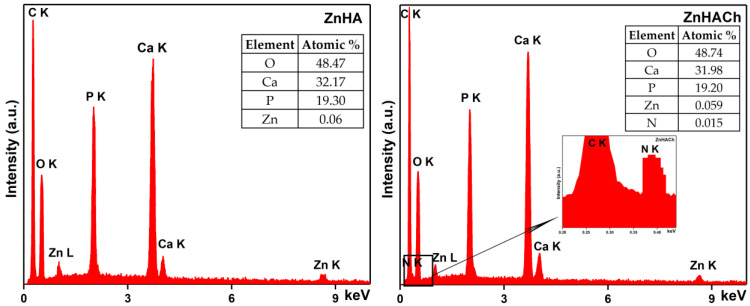
Energy dispersive X-ray spectroscopy (EDX) analysis of ZnHA and ZnHACh samples.

The chemical composition of the ZnHA and ZnHACh samples was studied with the aid of EDX analysis, and the results are shown in Figure 4. For the ZnHA powders, the presence of phosphorus (P), calcium (Ca), zinc (Zn) and oxygen (O) can be observed in the EDX spectrum (Figure 4). The carbon (C) line observed in Figure 4 is owed to the presence of double-adhesive carbon tape on which the powders were placed before the SEM analyses. In the case of the ZnHACh sample, the carbon (C) line appears in Figure 4 (left) due to the double-adhesive carbon tape and, on the other hand, due to the presence of chitosan in the studied sample. Furthermore, the presence of chitosan in the ZnHACh powders is also underlined by the presence of the nitrogen (N) line in their specific EDX spectra. In Figure 4 (inset) are presented the results of EDX quantitative analysis.

The other lines that are noticed in the EDX spectra of ZnHACh powders belong to the other four chemical elements (calcium, phosphorus, oxygen and zinc) that are specific to ZnHApCh composition. Finally, the results of the studies conducted by energy dispersive x-ray spectroscopy reveal the purity of the obtained samples.

**Figure 5:**
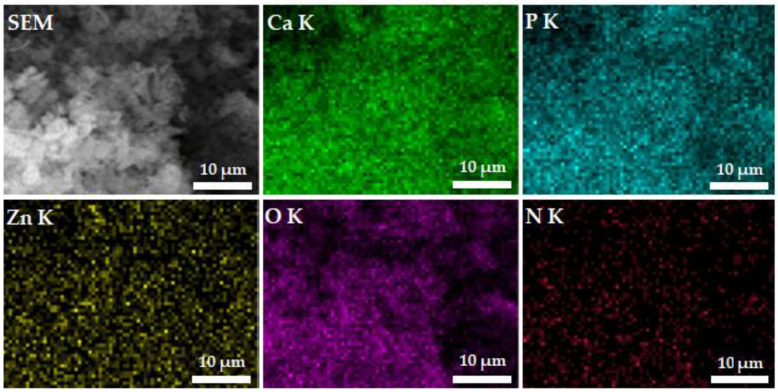
Elemental mapping of ZnHACh powder sample.

Figure 5 shows the SEM image of ZnHACh with the results of chemical mapping made using SEM. The results of elemental mapping highlight the well spatial distribution of phosphorus (P), calcium (Ca), nitrogen (N), oxygen (O) and zinc (Zn) in the ZnHACh powders. As can be seen in the Figure 5, the mapping images obtained on the studied samples highlight a homogeneous distribution of the main constituents (P, Ca, N, O and Zn).

**Figure 6:**
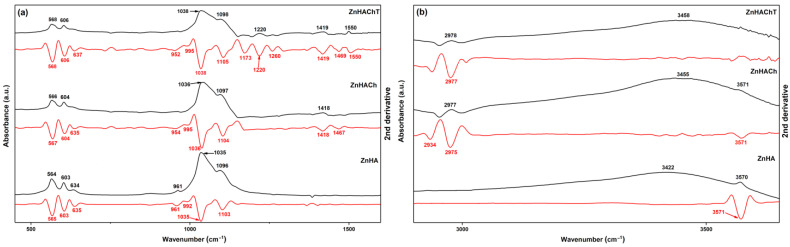
FTIR spectra (black line) and second derivative spectra (red line) characteristic to ZnHA, ZnHACh and ZnHAChT powders in 450–1600 cm^−1^ (**a**) and 2900–3650 cm^−1^ (**b**) spectral regions.

The results of FTIR spectral analysis (FTIR spectra and the second derivative spectra in the 450–3650 cm^−1^ spectral region) conducted on ZnHA, ZnHACh and ZnHAChT powders are presented in Figure 6.

In Figure 6, the black line represents the FTIR spectra and the red line represents the second derivative spectra obtained for ZnHA, ZnHACh and ZnHAChT samples. Therefore, the FTIR spectra of ZnHA samples is dominated mainly by the bands that are assigned to the vibrational modes of carbonate, phosphate or hydroxyl groups from the hydroxyapatite (HA) structure. The main bands associated to the bending mode of PO_4_^3−^ groups are found at 564 cm^−1^ and 603 cm^−1^ [8,36,37]. The main vibrational band observed at around 634 cm^−1^ belongs to the OH^−^ librational mode [8]. At around 961 cm^−1^ a maximum that may be attributed to ν_1_ vibration of PO_4_^3−^ groups [8,36,37] can be noticed. Moreover, in the 1035–1098 cm^−1^ spectral domain peaks characteristic to PO_4_^3−^ groups’ vibration from the HA structure are present [8,36,37]. At around 873 cm^−1^ could be observed a shoulder specific to the PO_4_^3−^ groups’ vibration [8,36,37]. In addition, for the ZnHACh sample, besides the presence of the specific vibration bands of ZnHAp presented above, the presence of maxima associated with the presence of chitosan in the 1300–1600 cm^−1^ spectral region is also observed [37]. The presence of the tarragon essential oil in the ZnHAChT powders is underlined by the appearance of new vibration bands (shoulder) around 1220 cm^−1^ and 1550 cm^−1^ that may belong to C-O and C=C stretching in estragole from tarragon essential oil [8,36,37,38,39,40]. Moreover, in the spectral region from 2900 to 3600 cm^−1^ (Figure 6b) can be noticed the presence of maxima that could be associated to the hydroxyl (from HA), amine and C-H vibrations (from chitosan). Therefore, at around 2977 cm^−1^ are observed broad maxima that may be attributed to the C-H asymmetric stretching in CH_3_ groups. The large maxima noticed (for all the studied samples) at about 3450 cm^−1^ are specific to the hydroxyl vibration from HA. For the ZnHACh and ZnHAChT samples, the broad maxima specific to the chitosan (N-H vibration) and hydroxyapatite (O-H vibrations) observed between 3300 and 3600 cm^−1^ are overlapped [41].

Furthermore, for a more compressive study of the molecular structure of obtained samples, the second derivative spectra were achieved in the conditions described elsewhere [41]. The red lines from Figure 6 represent the second derivative spectra of ZnHA, ZnHACh and ZnHAChT samples. Between 450 and 1600 cm^−1^ can be observed the maxima that may be attributed to the phosphate groups’ vibration (ν_1_, ν_3_ and ν_4_) [17]. Between 560 and 630 cm^−1^ the maxima that belong to ν_4_ of phosphate group are noticed, and between 1000 and 1100 cm^−1^ the presence of maxima associated to the ν_3_ vibration of phosphate group is highlighted [41]. Finally, the presence of ν_1_ vibration (of phosphate group) is highlighted by the maximum from 961 cm^−1^. Furthermore, in the case of the second derivative spectra of ZnHACh and ZnHAChT can be noticed the presence of maxima that belong to vibrations specific to chitosan (1300–1600 cm^−1^, 2934–2977 and around 3571 cm^−1^) [41] and to tarragon essential oil (at about 1220 cm^−1^ and 1550 cm^−1^) structure [38,39,40]. The presence of vibration bands associated with the hydroxyl groups can be noticed at around 637 cm^−1^ and around 3450 cm^−1^ for all the studied samples [8]. It is also observed that many of the bands specific to the tarragon essential oil structure are overlapped with those of chitosan and even with those of hydroxyapatite. More than that, the presence of chitosan and tarragon essential oil in the samples induce changes in the FTIR spectra. Therefore, it can be noticed that in the case of ZnHACh and ZnHAChT powders, the vibrational bands are wider and less intense compared to those of the ZnHA sample. These features are more pronounced in the case of the ZnHAChT sample. These characteristics of the FTIR spectra obtained for the ZnHACh and ZnHAChT powders suggest that the crystallinity of the ZnHA decreased due to the presence of chitosan and tarragon essential oil in the powders. As can be seen, the results of the FTIR spectral analysis are in concordance with the results of the XRD measurements. Similar behavior (the decrease of the band intensity and their broadening were underlined in the XRD and FTIR spectra) was reported by Danilchenko S.N. et al. [37] in their study entitled “Chitosan–hydroxyapatite composite biomaterials made by a one step co-precipitation method”. All these aspects suggest a decrease in the size of the nanoparticles and, at the same time, a decrease in the degree of crystallinity of the samples due to the chitosan/tarragon essential oil presence in the samples.

Together with the surface, the size of the crystallites have an important role in terms of the response to the bioactivity of the material. Previous studies [42,43] have shown that materials that are less crystalline are more bioresorbable and increase the functions of osteoblasts.

In recentyears, due to the increase in pathological conditions of bones, tissue engineering and regenerative medicine were highly employed in the treatment of these conditions [44,45,46,47,48,49,50]. For this purpose, over the years, extensive studies were undergone regarding the employment of biocompatible and bioresorbable materials with enhanced biological properties and having controllable degradation and resorption properties in the development of new devices for bone treatment [45,48]. Nowadays, data shows that osteoporosis is a chronic disease that has a strong impact on the general population and is characterized by compromised bone strength that leads to structural changes of the bone that increase the probability of fractures [51,52,53,54]. Moreover, even though artificial bone replacement has been used for decades in the treatment of bone defects, the use of prostheses can pose challenges such as infection, aseptic loosening or rejection. Therefore, the research regarding the fabrication of new materials with enhanced biological performance are of great need in order to overcome these challenges [55,56]. Infections associated with implantable devices are one of the main reasons for implant failure and could be caused either by an improper surgical performance or due to the contamination of the surrounding tissues [57]. In this context, the biological characteristics of the ZnHA, ZnHACh and ZnHAChT samples were studied.

**Figure 7:**
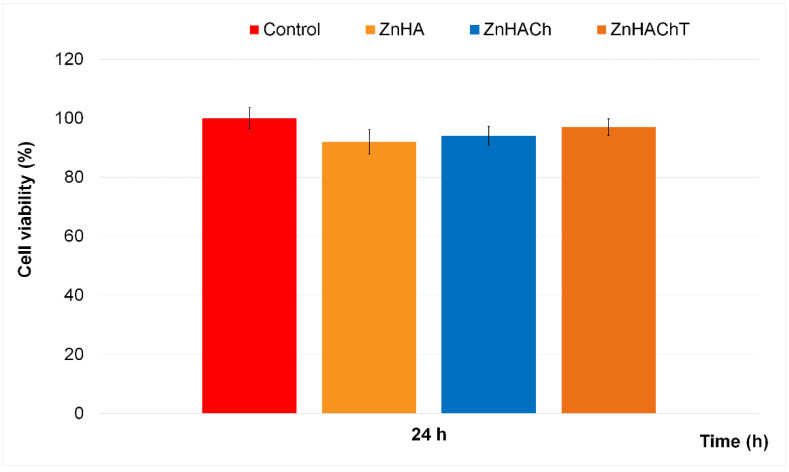
Graphical representation of the MTT assay of the cell viability of hFOB 1.19 cells after 24 h of incubation with ZnHA, ZnHACh and ZnHAChT.

The cytotoxicity of the ZnHA, ZnHACh and ZnHAChT was determined using human fetal osteoblast hFOB 1.19 cells by performing in vitro studies. For this purpose, hFOB 1.19 cell suspensions were exposed to the samples for 24 h, and their viability was determined employing the well-known MTT assay. Therefore, the results of the in vitro MTT assays regarding the cell viability of the hFOB 1.19 exposed for 24 h to ZnHA, ZnHACh and ZnHAChT are depicted in Figure 7. The in vitro experiments were done in triplicate and the data was presented as mean ± Standard Deviation (S.D.). Thus, the results of the MTT studies highlighted that all the investigated biocomposites exhibited strong biocompatible properties after 24 h.

The MTT assay revealed that after 24 h of exposure to the ZnHA, ZnHACh and ZnHAChT samples, the cell viability of the hFOB 1.19 cells was maintained above 92% compared to the control, which emphasizes that the biocomposites present very good biocompatible properties towards hFOB 1.19 cells. Moreover, the results also suggested that the presence of both chitosan and tarragon essential oil as well as the synergies involved in the composite samples contributed to an increase of the cell viability compared to the ZnHA sample. The results are in good concordance with earlier reported studies about the biological properties of hydroxyapatite and hydroxyapatite-based composites [8,58,59,60].

**Figure 8:**
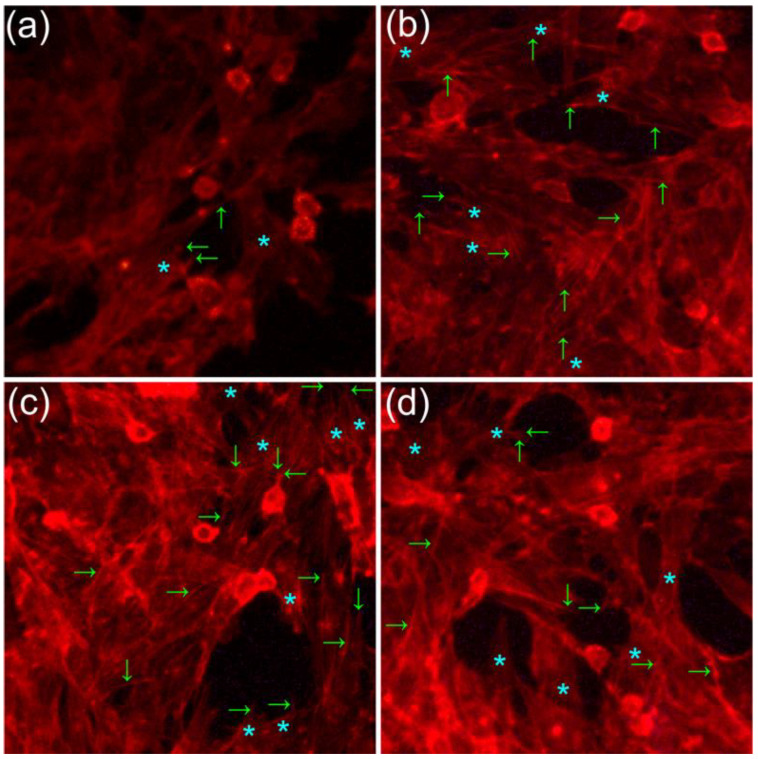
Fluorescence micrographs of primary human osteoblast cells (hFOB 1.19) incubated with ZnHA (**b**), ZnHACh (**c**) and ZnHAChT (**d**) for 24 h comparative to an untreated hFOB 1.19 cell culture used as control (**a**); green arrows indicate the presence of filopodia and, for the light blue asterisk, the presence of lamelipodia.

Complex information was also gathered by studying the morphology of the hFOB 1.19 cells exposed for 24 h to ZnHA, ZnHACh and ZnHAChT. The fluorescence microscopy of the hFOB 1.19 cells exposed for 24 h to ZnHA, ZnHACh and ZnHAChT are depicted in Figure 8. The visualization of the hFOB 1.19 cells after 24 h of exposure to ZnHA, ZnHACh and ZnHAChT emphasized that the morphology of the cells was not altered by the presence of the samples. More than that, the fluorescence micrographs also depicted that after 24 h of exposure to the ZnHA, ZnHACh and ZnHAChT biocomposites, the cells still exhibited the normal typical morphology of hFOB 1.19 cells. The results are in agreement with the results provided by the MTT assays which showed that the exposure to the ZnHA, ZnHACh and ZnHAChT for 24 h did not exhibit any toxic effect against the viability of hFOB 1.19 cells. In addition, the fluorescence microscopy images also determined that the investigated samples promoted the proliferation and cell development of hFOB 1.19 cells and also emphasized that the samples favored the cells’ spread and their evolution into a layer.

More than that, the results highlighted that the presence of chitosan and tarragon essential oil in the composite materials promoted the cell development and proliferation of hFOB 1.19.

Furthermore, the fluorescence micrographs also showed the presence of both lamellipodia and filopodia, depicted in Figure 8 by green arrows (filopodia) and light blue asterisks (lamellipodia), which strengthen the conclusion that ZnHA, ZnHACh and ZnHAChT possess good biocompatible properties. Usually, the development and proliferation of cells are determined by various parameters such as size and shape of the particles, surface chemistry, roughness, surface chemistry, etc. [61,62,63]. The initial cell attachment is mediated by thread-like appendages, called filopodia, that extend several micrometers from the cell body to explore the extracellular surface and have the role of anchoring the cell. Filopodia was firstly described in living cells in 1961 by Gustafson and Wolpert [64] and since then have been considered one of the cell’s main sensory tools. The presence of lamellipodia and filopodia are strongly correlated with the quality response that cells exhibit to the materials and surface that they were exposed to. Therefore, the presence and extension of numerous lamellipodia and filopodia are typically characteristic of good biocompatibility properties. The results of the cytotoxicity assays as well as the microscopic visualization of the hFOB 1.19 cells exposed for 24 h to ZnHA, ZnHACh and ZnHAChT samples determined that the materials possess high biocompatible properties and that their presence did not induce any morphological changes in the structure of the cells. These results are in concordance with earlier reported data about the biocompatibility of zinc-doped hydroxyapatite composites materials [65,66,67,68]. In their study, Korbut et al. [50], regarding the “three component composite scaffolds based on PCL, hydroxyapatite, and L-lysine obtained in TIPS-SL: bioactive material for bone tissue engineering”, showed that the modification of the scaffolds using hydroxyapatite and L-Lysine conduced to the enhancement of the regenerative potential of the composites and also to an increase of the adhesion of the human osteoblast cells. Moreover, studies conducted by Thian et al. [65] highlighted that the biological properties of hydroxyapatite are influenced by the presence of Zn^2+^ ions in the HA structure. Zinc is a trace element that is actively involved in numerous metabolic processes, and which also activates proteins involved in bone homeostasis [50,68,69,70,71]. Zinc is present in a proportion of approximately 30% in bones and plays a key role in bone formation [50,68,69,70,71], and bone growth is influenced by the zinc levels present in the bones. The studies performed by Shitole et al. [72] determined that the incorporation of nano-HA and ZnO-NPs into the PCL scaffold greatly improved their osteogenic properties. Moreover, Maimaiti et al. [73] reported that a HA/Zn coating exhibited superior osteogenic properties compared to a pure HA coating. The preliminary results obtained regarding the cytotoxicity of the ZnHA, ZnHACh and ZnHAChT, which correlated with previously reported results, could guide the future development of novel strategies for the obtaining of materials with superior osteogenic activity and improved cytobiocompatibility.

**Figure 9:**
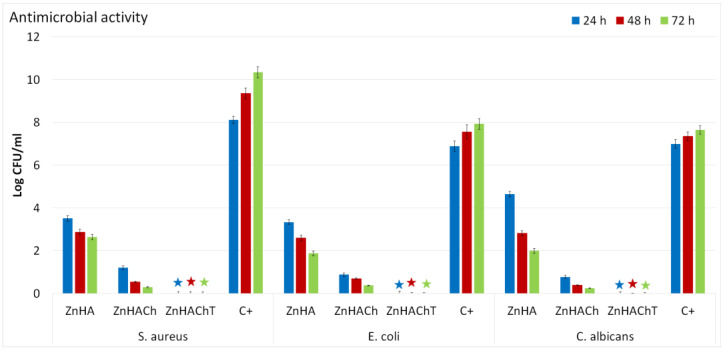
Graphical representation of the antimicrobial activity of ZnHA, ZnHACh and ZnHAChT after 24, 48 and 72 h of exposure to *Staphylococcus aureus* ATCC 25923, *Escherichia coli* ATCC 25922 and *Candida albicans* ATCC 10231.

In recent years, due to an increase of bone disease affections, mainly caused by population aging and poor medical management of these affections, the incidence of severe cases of infections has increased considerably. The occurrence of implant-related infections is the principal cause of implant failure, and can occur either because of an improper surgical procedure or because of post-operation contamination [74,75,76], and could lead to great financial burdens to patients, and can cause life-threateningconditions affecting the quality of life of patients. Therefore, in the last years, there have been tremendous efforts to inhibit at the early stage the microorganism’s attachment and proliferation, by developing new materials with antimicrobial properties that might be used as implant coatings. In this context, our study also involved the evaluation of the antimicrobial properties of ZnHA, ZnHACh and ZnHAChT composite materials. The antimicrobial activity of the ZnHA, ZnHACh and ZnHAChT were evaluated against *Escherichia coli* ATCC 25922, *Staphylococcus aureus* ATCC 25923 and *Candida albicans* ATCC 10231 microbial strains. For this aim, the samples were put into contact with the microbial suspensions and their antimicrobial effects were determined after 24, 48 and 72 h. The results of the antimicrobial assays are presented in Figure 9. The data obtained from the antimicrobial assays depicted that all the samples inhibited the development of the microbial strains even after 24 h of incubation. At the same time, the results suggested that the presence of chitosan and tarragon essential oil improved significantly the antimicrobial effects of the biocomposites against the tested microorganisms. The results of the in vitro antimicrobial assays highlighted that the antimicrobial activity of the samples was affected by the incubation time as well as the tested microbial strain. The data showed that the best inhibitory effects were present after 72 h of incubation for both ZnHA and ZnHACh samples. More than that, the results highlighted that the presence of chitosan increased the antimicrobial activity of ZnHA samples.

Furthermore, the results depicted that the presence of chitosan and tarragon essential oil conferred the sample bactericidal and fungicidal effects being able to completely eradicate the microbial strains for all the tested time intervals. The results reported in this paper are in concordance with early reported studies regarding the antimicrobial properties of composite materials and coatings based on hydroxyapatite, zinc ions, chitosan and tarragon essential oils [77,78,79,80,81,82]. Even though antimicrobial materials have been extensively studied, the exact mechanism responsible for their antimicrobial activity are not yet fully understood. Moreover, the antimicrobial properties of materials are attributed to the properties of their chemical constituents as well as the synergies that appear between them. In addition, the antimicrobial activity is also influenced by numerous parameters such as the type of the microorganism, the shape and size of the particles, the surface chemistry, the state of the material, etc. Furthermore, the metabolism and species of bacteria can also strongly affect the antimicrobial behaviors of various materials. In the case of the ZnHA, ZnHACh and ZnHAChT composites, the antimicrobial properties could be ascribed to the presence of zinc ions, chitosan and tarragon essential oil as well as the synergies that appear in the composite materials due to their constituents. In the case of zinc-doped hydroxyapatite materials, it is well known that zinc ions play an important role in their antimicrobial properties due to the fact that Zn^2+^ could interfere with cell membrane permeability by combining with functional proteins. If the Zn^2+^ concentration increases in the intracellular space, the interaction that takes place with the thiol group of the enzyme is increased. This phenomenon could disturb the bacterial enzymatic reactions, thus weakening glycolysis and could induce bacterial cell death [83]. Chitosan is a well-known natural polymer that has been proven to possess antimicrobial properties as well as bactericidal activity and was deemed by the United States Food and Drug Administration (FDA) as being GRAS (Generally Recognized as Safe by FDA) [82,84,85]. One of the principal mechanisms deemed to be responsible for the antimicrobial activity of chitosan has been reported as being rendered by the fact that the positive charges resulting from the amino groups of chitosan could interact electrostatically with the negatively charged components of the microbial membranes [86]. In addition to this, other mechanisms by which chitosan molecules could inhibit the growth of microorganisms were also described. One of the accepted possible mechanisms is the ability of chitosan to bind to trace elements and to actively interrupt the nutrients necessary for the growth of microbial cells. Another accepted mechanism insinuates that chitosan molecules could pass through the wall of microbial cells and suppress the synthesis of mRNA by binding to the DNA [87,88]. Furthermore, the antimicrobial properties of plants and plant extracts as well as essential oils are not usually attributed to an individual constituent, but rather to the interaction of the compounds present in their composition. There are reports that indicated there is evidence that the antimicrobial activity of extracts is attributed to the fact that they could make changes to the structure and function of the cell membrane. Studies revealed that extracts and essential oils have the ability to increase the microbial cell’s membrane diffusivity, eventually leading to cell death [89]. The mechanism by which essential oil have the ability to inhibit the growth of microorganisms include different modes of action and are mainly attributed to their hydrophobicity. Because of their hydrophobicity, they could easily pass into the lipid bilayer of the microbial cell’s membrane, making it more permeable, which usually leads to a leakage of vital cell contents and eventually to cell death [90,91]. Furthermore, another potential antimicrobial mechanism of essential oils was attributed to the fact that the essential oils have the ability to disrupt bacterial enzyme systems [90,91]. In addition, the antimicrobial activity of the essential oils is also defined by their chemical constituents. Essential oils are complex natural mixtures composed of about 20–60 chemical constituents with different concentrations [91]. Usually, essential oils are characterized by two or maybe three major chemical components found in high concentrations (approx. 20–70%) compared to the other chemical components that are present only in trace amounts [91]. Generally, biological properties of the essential oils are determined by their major chemical constituents [92,93]. In the case of our study, the essential oil used to prepare ZnHAChT was French tarragon (*Artemisia dracunculus* L.) and the major chemical constituents of tarragon essential oil were reported to be estragole, sabinene, methyleugenol, *trans*-anethole, limonene, terpinolene,β-ocimene, terpinen-4-ol, α-ocimene and elemicin [19,94,95,96]. Estragole, also known as methyl chavicol, has been reported to exhibit a broad spectrum of antifungal activities against *Mucor mucedo*, *Aspergillus niger*, *Botryodiplodia theobromae*, Fusarium solani, *Candida albicans*, *Rhizopus solani* and *Microsporum gypseum* which were observed to be achieved by causing the disruption of fungal membrane [97]. In the study conducted by Petrosyan et al. [98], the data showed that the tarragon essential oils exhibited stronger antimicrobial effects against gram-positive bacteria and yeasts than against gram-negative ones. Furthermore, in their studies, Raeisi et al. [99] reported that *S. aureus* is more sensitive than *E. coli* to tarragon essential oil. Reza Sharafati Chaleshtori et al. [100], in their studies about the “the evaluation of the antibacterial and antioxidant activity of tarragon (*Artemisia Dracunculus* L.) essential oil and its chemical composition”, determined that the essential oil exhibited different degrees of inhibitory activity against the growth of the tested bacterial strains. Their study concluded that bacterial strains belonging to gram-negative bacteria, especially *Sh. dysenteriae* and *S. marcescens,* were the most sensitive, while *S. aureus* and *L. monocytogenes*, belonging to gram-positive bacteria, were the most resistant against this essential oil [100]. The preliminary results obtained in our study are in good agreement with previously reported data and also strengthen the hypothesis that the antimicrobial mechanisms of the samples could be influenced both by the constituent chemical elements as well as by the synergies that appear in the composite material. More than that, even if further future complex in vitro and in vivo studies as well as clinical trials are needed to verify the prospective use of composite materials based on hydroxyapatite, zinc, chitosan and tarragon essential as antimicrobial agents, our findings are encouraging and could represent an important stepping stone in the future development of new antimicrobial agents with reduced toxicity, specific antimicrobial mechanism and good osteogenic properties.

## 4. Conclusions

In this work, we report the synthesis of new biocomposite materials based on zinc-doped hydroxyapatite/chitosan enriched with essential oil of *Artemisia dracunculus* L. The properties of the obtained samples were investigated using scanning electron microscopy (SEM), X-ray diffraction (XRD), Fourier transform infrared spectroscopy (FTIR) and energy dispersive X-ray spectroscopy (EDX). The XRD results revealed the presence of HA in the samples. The nanometric dimension of the ZnHA was highlighted by the results of both XRD and SEM studies. The purity of the analyzed specimens was demonstrated by the results of EDX analyses. The maxima found in the FTIR spectra belong mainly to the vibration characteristic to the functional groups from HA, chitosan and tarragon essential oil structure.

The cytotoxic activity of the ZnHA, ZnHACh and ZnHAChT was assessed with the aid of the hFOB 1.19 cell line. The results of the MTT assays depicted that the in vitro cell viability was above 92% and reached as high as 97% after 24 h of exposure to the ZnHA, ZnHACh and ZnHAChT. Moreover, the fluorescence micrographs also showed that the samples did not induce any morphological changes in the hFOB 1.19 cells after 24 h of exposure. Furthermore, the fluorescence micrographs showed that after 24 h of exposure to the ZnHA, ZnHACh and ZnHAChT samples, the presence of lamellipodia and filopodia was observed which is characteristic to a good biocompatibility of the tested sample. In addition, the antimicrobial activity of the ZnHA, ZnHACh and ZnHAChT was also evaluated against *Escherichia coli* ATCC 25922, *Staphylococcus aureus* ATCC 25923 and *Candida albicans* ATCC 10231. The results of the antimicrobial assays depicted that all the samples own a strong inhibitory effect against all the tested microbial strains for all the tested time intervals. Moreover, the data suggested that the antimicrobial activity was influenced by both the incubation time as well as the tested microbial strains and the sample. The results highlighted that in the case of ZnHApChT, a bactericidal and fungicidal effect was achieved. These results are promising data for the future development of novel composite materials with enhanced biological properties that could promote the osteogenic process of bone healing and also exhibit antimicrobial properties.

## Data Availability

Not applicable.
